# Supplementary material for: Synthesis, Crystal Structure, and Luminescence of Cadmium(II) and Silver(I) Coordination Polymers Based on 1,3-Bis(1,2,4-triazol-1-yl)adamantane
Source: Molecules. 2021 Sep 5;26(17):5400. doi: 10.3390/molecules26175400 (PMC8434004; doi:10.3390/molecules26175400)

checkCIF/PLATON report

Structure factors have been supplied for datablock(s) pot_cd008_1

THIS REPORT IS FOR GUIDANCE ONLY. IF USED AS PART OF A REVIEW PROCEDURE FOR PUBLICATION, IT SHOULD NOT REPLACE THE EXPERTISE OF AN EXPERIENCED CRYSTALLOGRAPHIC REFEREE.

No syntax errors found. CIF dictionary Interpreting this report

Datablock: pot_cd008_1

```
Bond precision:  C-C = 0.0132 A          Wavelength=0.71073

Cell:           a=14.4331(6)    b=12.1592(6)       c=25.3803(12)
                alpha=90        beta=104.848(2)    gamma=90
Temperature:    298 K
```

	Calculated	Reported
Volume	4305.4(3)	4305.4(3)
Space group	P 21/c	P 1 21/c 1
Hall group	-P 2ybc	-P 2ybc
Moiety formula	2(C15 H22 Cd N8 O7), C H4 O	C15 H22 Cd N8 O7, 0.5(C H4 O)
Sum formula	C31 H48 Cd2 N16 O15	C15.50 H24 Cd N8 O7.50
Mr	1109.67	554.83
Dx,g cm-3	1.712	1.712
Z	4	8
Mu (mm-1)	1.073	1.073
F000	2248.0	2248.0
F000’	2242.50	
h,k,lmax	17,14,30	17,14,30
Nref	7607	7600
Tmin,Tmax	0.793,0.928	0.648,0.745
Tmin’	0.765	

```
Correction method= # Reported T Limits: Tmin=0.648 Tmax=0.745
AbsCorr = MULTI-SCAN

Data completeness= 0.999          Theta(max)= 25.026

R(reflections)= 0.0724( 5278)     wR2(reflections)= 0.1631( 7600)

S = 1.168                  Npar= 587
```

```
The following ALERTS were generated. Each ALERT has the format
       test-name_ALERT_alert-type_alert-level.
Click on the hyperlinks for more details of the test.
```

Alert level C

```
PLAT232_ALERT_2_C Hirshfeld Test Diff (M-X)  Cd1      --O31      .        5.6 s.u.
PLAT232_ALERT_2_C Hirshfeld Test Diff (M-X)  Cd2      --O51      .        5.2 s.u.
PLAT241_ALERT_2_C High   'MainMol' Ueq as Compared to Neighbors of        O31 Check
PLAT241_ALERT_2_C High   'MainMol' Ueq as Compared to Neighbors of        O32 Check
PLAT241_ALERT_2_C High   'MainMol' Ueq as Compared to Neighbors of        O41 Check
PLAT241_ALERT_2_C High   'MainMol' Ueq as Compared to Neighbors of        O42 Check
PLAT241_ALERT_2_C High   'MainMol' Ueq as Compared to Neighbors of         O8 Check
PLAT241_ALERT_2_C High   'MainMol' Ueq as Compared to Neighbors of        O51 Check
PLAT241_ALERT_2_C High   'MainMol' Ueq as Compared to Neighbors of        O52 Check
PLAT241_ALERT_2_C High   'MainMol' Ueq as Compared to Neighbors of        O62 Check
PLAT242_ALERT_2_C Low    'MainMol' Ueq as Compared to Neighbors of        Cd1 Check
PLAT242_ALERT_2_C Low    'MainMol' Ueq as Compared to Neighbors of         N3 Check
PLAT242_ALERT_2_C Low    'MainMol' Ueq as Compared to Neighbors of         N4 Check
PLAT242_ALERT_2_C Low    'MainMol' Ueq as Compared to Neighbors of        Cd2 Check
PLAT242_ALERT_2_C Low    'MainMol' Ueq as Compared to Neighbors of         N5 Check
PLAT242_ALERT_2_C Low    'MainMol' Ueq as Compared to Neighbors of         N6 Check
PLAT342_ALERT_3_C Low Bond Precision on  C-C Bonds ...............    0.01321 Ang.
PLAT906_ALERT_3_C Large K Value in the Analysis of Variance ......     16.972 Check
PLAT906_ALERT_3_C Large K Value in the Analysis of Variance ......      3.519 Check
PLAT911_ALERT_3_C Missing FCF Refl Between Thmin & STh/L=    0.595          8 Report
PLAT934_ALERT_3_C Number of (Iobs-Icalc)/Sigma(W) > 10 Outliers ..          1 Check
PLAT975_ALERT_2_C Check Calcd Resid. Dens.  0.95A   From O53             0.45 eA-3
```

Alert level G

```
PLAT002_ALERT_2_G Number of Distance or Angle Restraints on AtSite          8 Note
PLAT003_ALERT_2_G Number of Uiso or Uij Restrained non-H Atoms ...          6 Report
PLAT004_ALERT_5_G Polymeric Structure Found with Maximum Dimension          1 Info
PLAT007_ALERT_5_G Number of Unrefined Donor-H Atoms ..............          1 Report
PLAT042_ALERT_1_G Calc. and Reported Moiety Formula Strings Differ     Please Check
PLAT045_ALERT_1_G Calculated and Reported Z Differ by a Factor ...       0.50 Check
PLAT083_ALERT_2_G SHELXL Second Parameter in WGHT  Unusually Large      47.34 Why ?
PLAT172_ALERT_4_G The CIF-Embedded .res File Contains DFIX Records          2 Report
PLAT173_ALERT_4_G The CIF-Embedded .res File Contains DANG Records          4 Report
PLAT186_ALERT_4_G The CIF-Embedded .res File Contains ISOR Records          1 Report
PLAT187_ALERT_4_G The CIF-Embedded .res File Contains RIGU Records          2 Report
PLAT232_ALERT_2_G Hirshfeld Test Diff (M-X)  Cd1      --O32      .        7.2 s.u.
PLAT432_ALERT_2_G Short Inter X...Y Contact  O43      ..C212             2.95 Ang.
                                                      x,y,z  =      1_555 Check
PLAT432_ALERT_2_G Short Inter X...Y Contact  O53      ..C7               3.01 Ang.
                                           1-x,-1/2+y,3/2-z  =      2_646 Check
PLAT432_ALERT_2_G Short Inter X...Y Contact  O63      ..C112             2.89 Ang.
                                                   -1+x,y,z  =      1_455 Check
PLAT720_ALERT_4_G Number of Unusual/Non-Standard Labels ..........          3 Note
PLAT794_ALERT_5_G Tentative Bond Valency for Cd1       (II)      .       2.16 Info
PLAT794_ALERT_5_G Tentative Bond Valency for Cd2       (II)      .       2.37 Info
PLAT860_ALERT_3_G Number of Least-Squares Restraints .............         33 Note
PLAT909_ALERT_3_G Percentage of I>2sig(I) Data at Theta(Max) Still        42% Note
PLAT933_ALERT_2_G Number of OMIT Records in Embedded .res File ...          4 Note
PLAT978_ALERT_2_G Number C-C Bonds with Positive Residual Density.          0 Info
```

---

 0 **ALERT level A** = Most likely a serious problem - resolve or explain
 0 **ALERT level B** = A potentially serious problem, consider carefully
22 **ALERT level C** = Check. Ensure it is not caused by an omission or oversight

```
22 ALERT level G = General information/check it is not something unexpected

 2 ALERT type 1 CIF construction/syntax error, inconsistent or missing data
26 ALERT type 2 Indicator that the structure model may be wrong or deficient
 7 ALERT type 3 Indicator that the structure quality may be low
 5 ALERT type 4 Improvement, methodology, query or suggestion
 4 ALERT type 5 Informative message, check
```

It is advisable to attempt to resolve as many as possible of the alerts in all categories. Often the minor alerts point to easily fixed oversights, errors and omissions in your CIF or refinement strategy, so attention to these fine details can be worthwhile. In order to resolve some of the more serious problems it may be necessary to carry out additional measurements or structure refinements. However, the purpose of your study may justify the reported deviations and the more serious of these should normally be commented upon in the discussion or experimental section of a paper or in the "special_details" fields of the CIF. checkCIF was carefully designed to identify outliers and unusual parameters, but every test has its limitations and alerts that are not important in a particular case may appear. Conversely, the absence of alerts does not guarantee there are no aspects of the results needing attention. It is up to the individual to critically assess their own results and, if necessary, seek expert advice.

Publication of your CIF in IUCr journals

A basic structural check has been run on your CIF. These basic checks will be run on all CIFs submitted for publication in IUCr journals (*Acta Crystallographica*, *Journal of Applied Crystallography*, *Journal of Synchrotron Radiation*); however, if you intend to submit to *Acta Crystallographica Section C* or *E* or *IUCrData*, you should make sure that full publication checks are run on the final version of your CIF prior to submission.

Publication of your CIF in other journals

Please refer to the *Notes for Authors* of the relevant journal for any special instructions relating to CIF submission.

PLATON version of 13/07/2021; check.def file version of 13/07/2021

Datablock pot_cd008_1 - ellipsoid plot

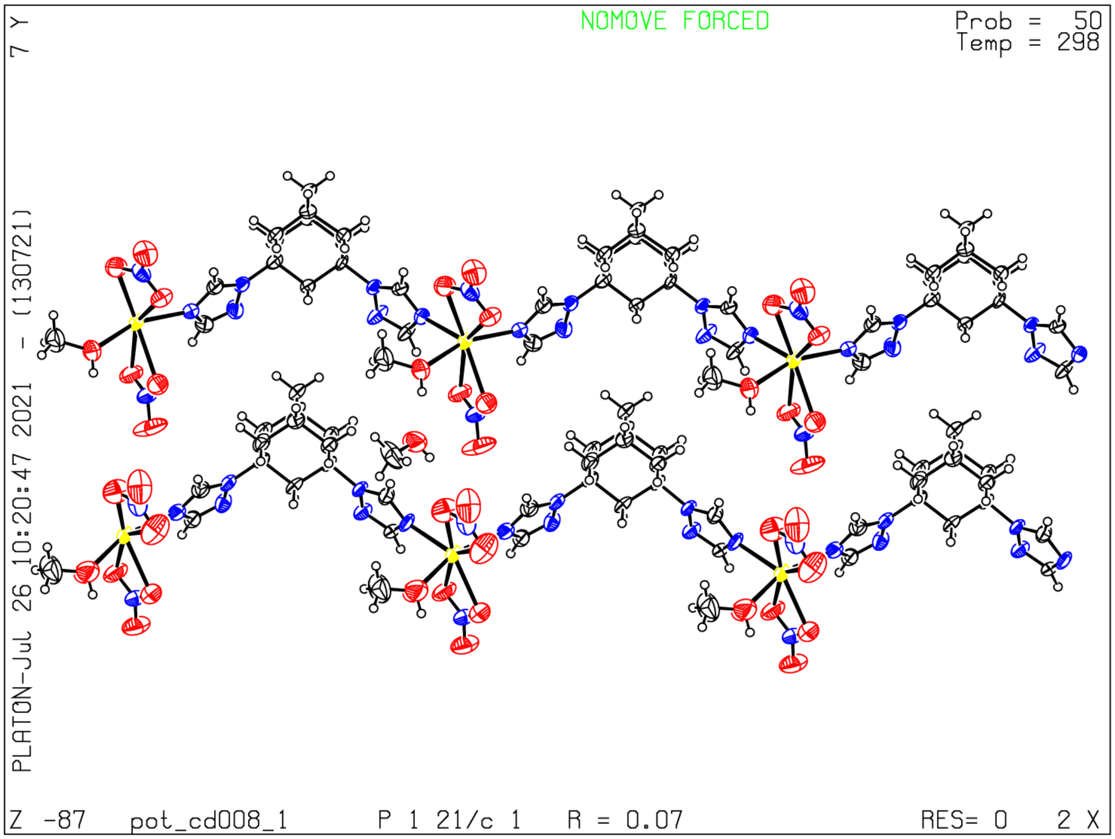

Supplement: Supplementary file 1 [file molecules-26-05400-s001.zip › Compound3_CheckCIF.pdf]
