# Supplementary material for: Synthesis, Crystal Structure, and Luminescence of Cadmium(II) and Silver(I) Coordination Polymers Based on 1,3-Bis(1,2,4-triazol-1-yl)adamantane
Source: Molecules. 2021 Sep 5;26(17):5400. doi: 10.3390/molecules26175400 (PMC8434004; doi:10.3390/molecules26175400)
Supplement: Supplementary file 1 [file molecules-26-05400-s001.zip › Tr2Adm_Cd_Ag_supporting.pdf]

Synthesis, crystal structure and luminescence of cadmium(II) and silver(I) coordination polymers based on 1,3-bis(1,2,4-triazol-1-yl)adamantane

Roman D. Marchenko [1], Taisiya S. Sukhikh [2], Alexey A. Ryadun [2] and Andrei S. Potapov [2,*]

[1] Kizhner Research Center, National Research Tomsk Polytechnic University, 30 Lenin Ave., 634050 Tomsk, Russia; rdm1@tpu.ru
[2] Nikolaev Institute of Inorganic Chemistry, Siberian Branch of the Russian Academy of Sciences, 3 Lavrentiev Ave., 630090 Novosibirsk, Russia; sukhikh@niic.nsc.ru (TS), ryadunalexey@mail.ru (AR), potapov@niic.nsc.ru (AP)
* Correspondence: potapov@niic.nsc.ru; Tel.: +7 (383) 330-94-90

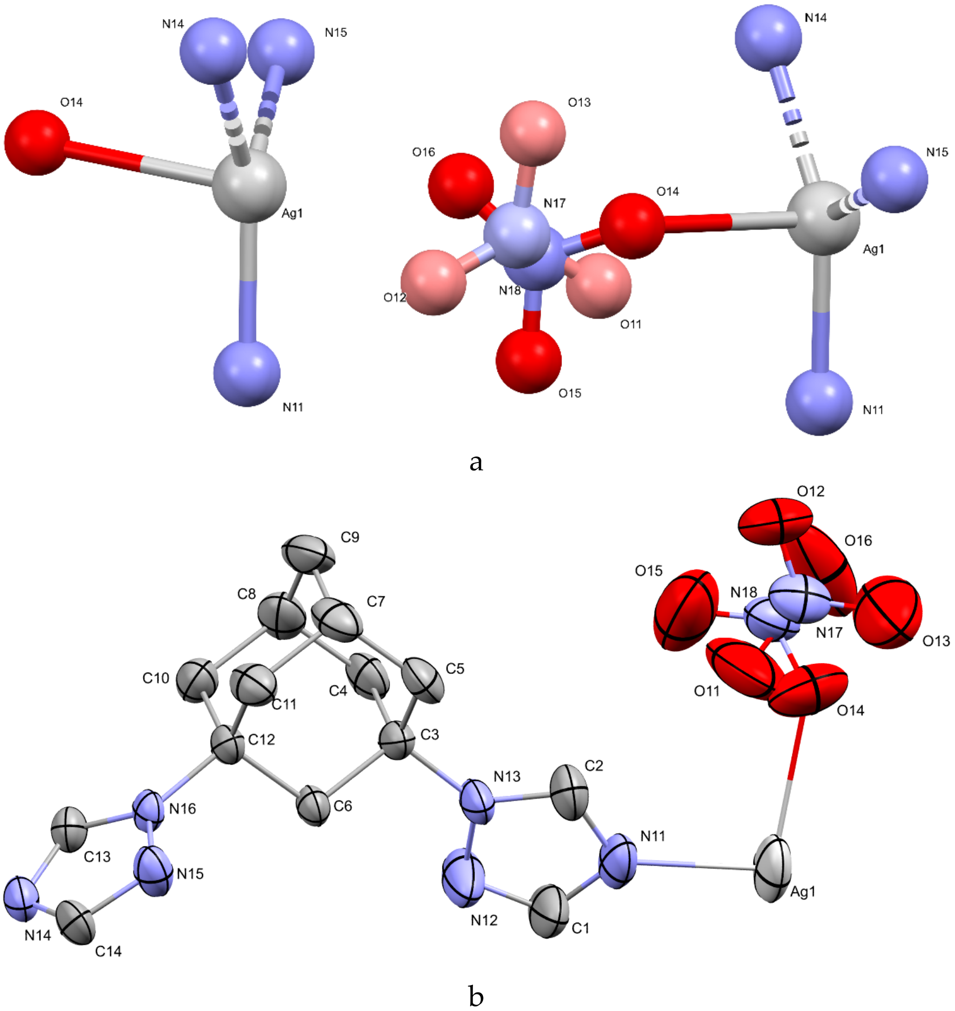

Figure S1. Disorder of nitrate ions in the structure of the coordination polymer **1** (a) and the asymmetric unit of the coordination polymer **1** showing both positions of the nitrate ions (b).

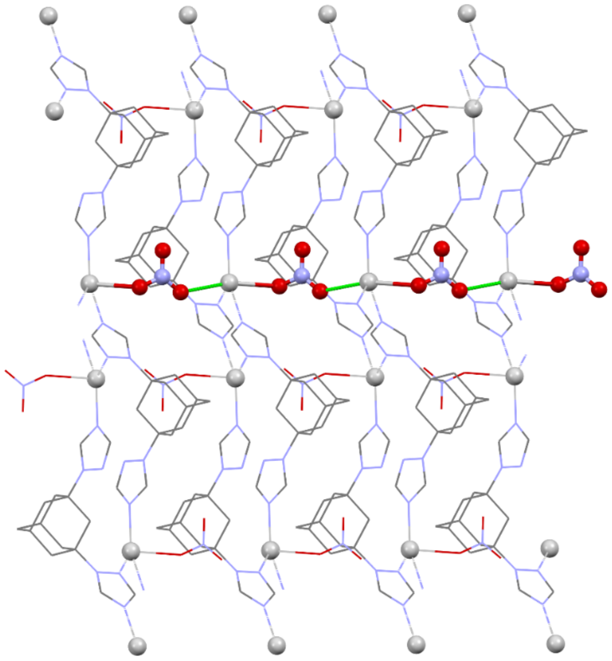

Figure S2. Bridging nitrate ions in the structure of the coordination polymer **1**. View along crystallographic axis *b*. Atom legend: Ag – grey balls; C – dark grey dots; N – blue balls and dots; O – red balls and dots.

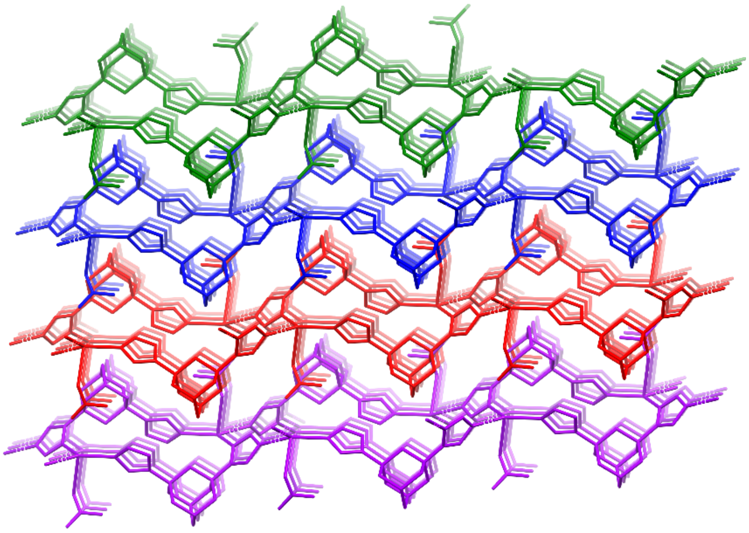

Figure S3. Packing of layers of the coordination polymer **1**. View along crystallographic axis *b*.

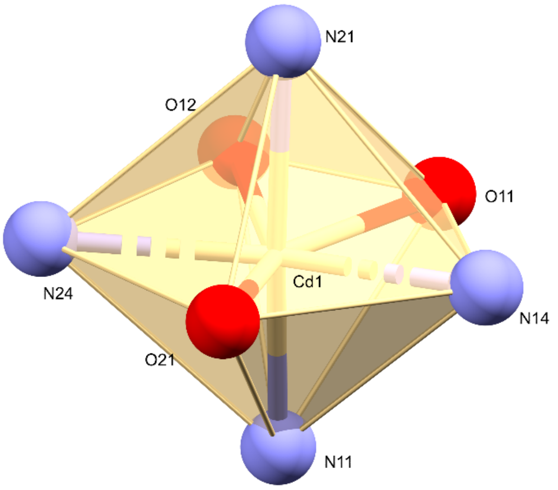

Figure S4. Coordination polyhedron of Cd(II) ion in the coordination polymer **2**.

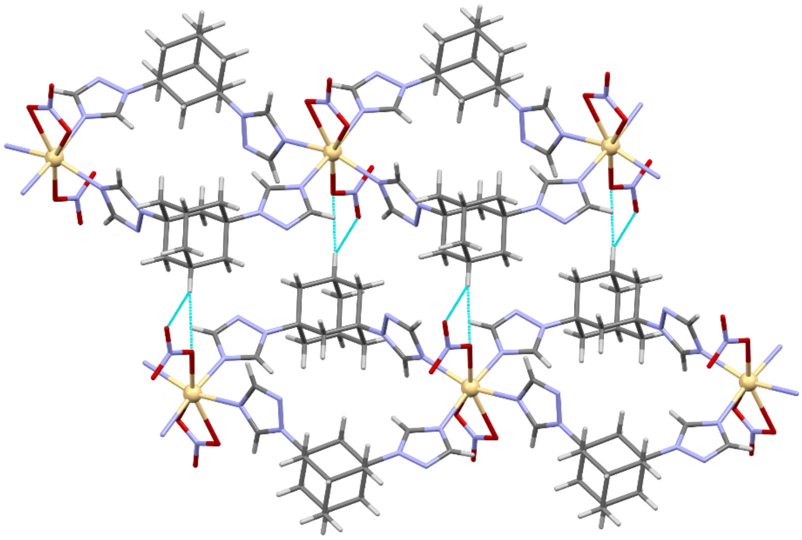

Figure S5. Close CH···O contacts between the chains of the coordination polymer **2**. Atom legend: Cd – yellow balls; C – dark grey dots; N – blue dots; O – red dots; H – white dots.

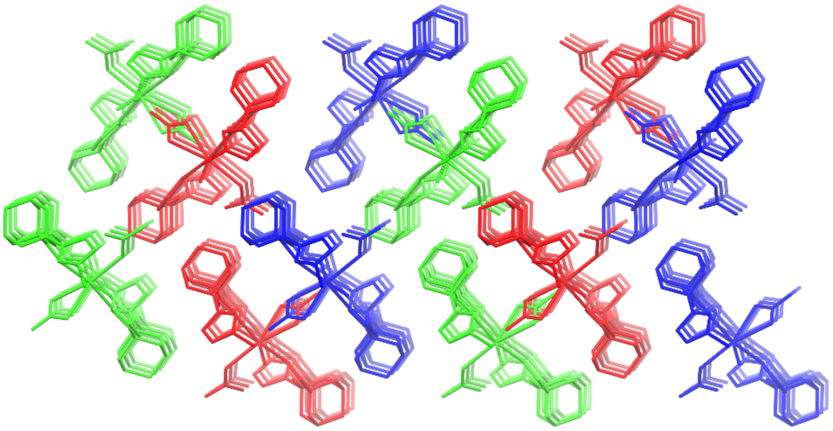

Figure S6. Packing of chains of the coordination polymer **1**. View along crystallographic axis *a*.

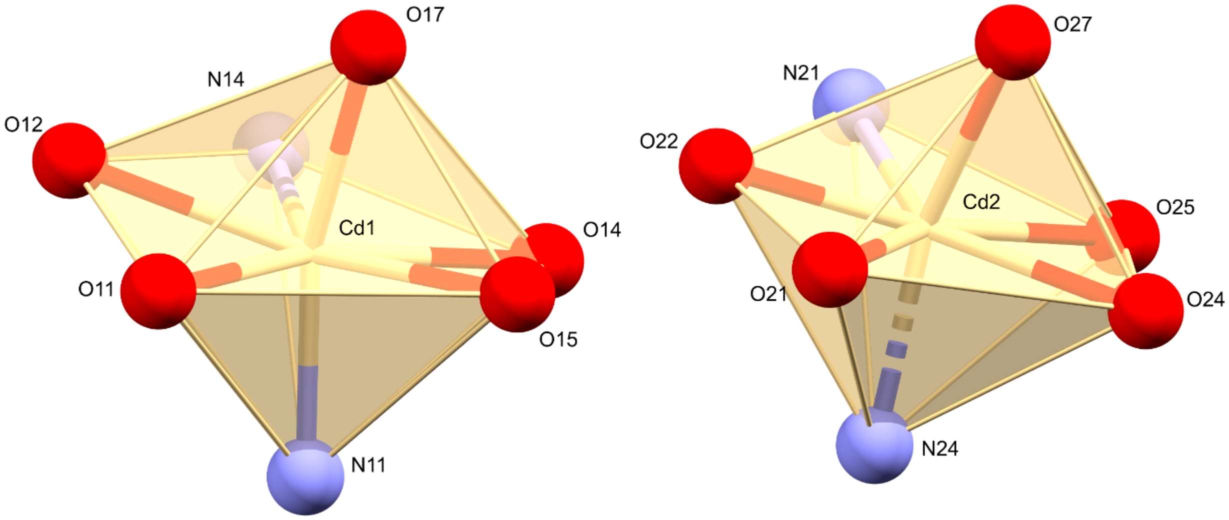

Figure S7. Coordination polyhedra of two crystallographically independent Cd(II) ions in the coordination polymer **3**.

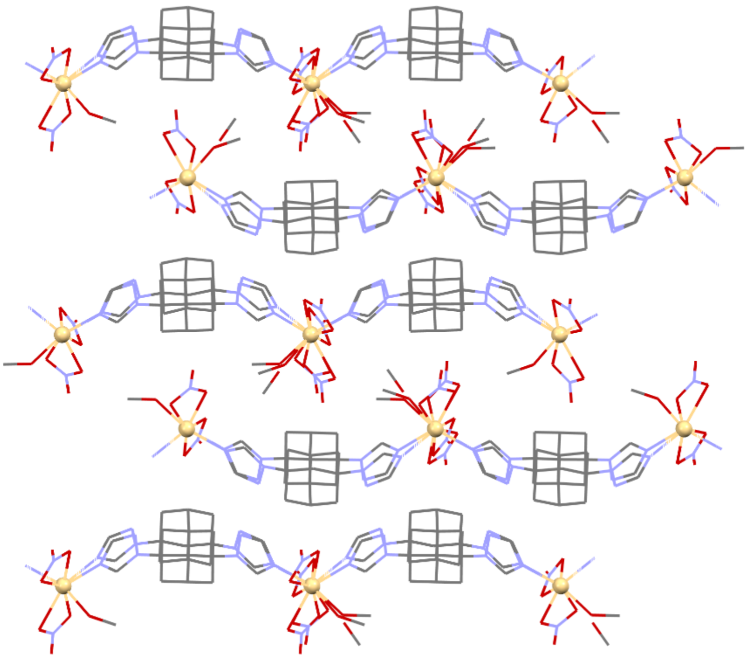

Figure S8. Packing of chains of the coordination polymer **3** into supramolecular layers. View along crystallographic axis *a*. Atom legend: Cd – yellow balls; C – dark grey dots; N – blue dots; O – red dots.

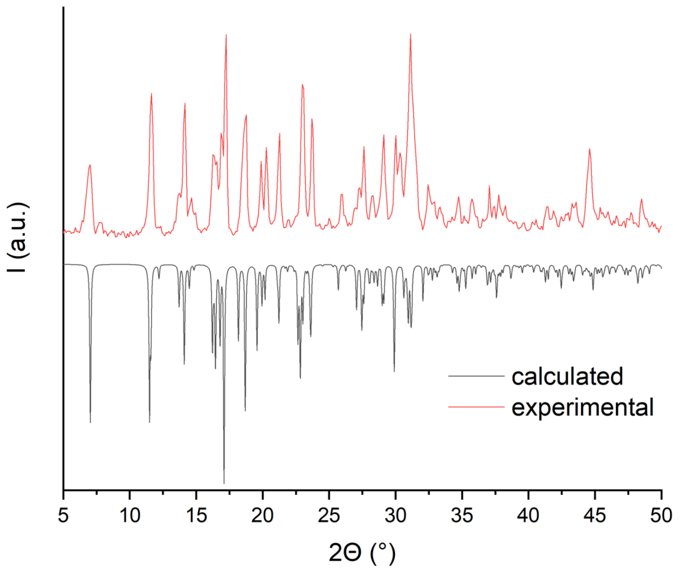

Figure S9. Powder X-ray diffraction plots for the coordination polymer **1**.

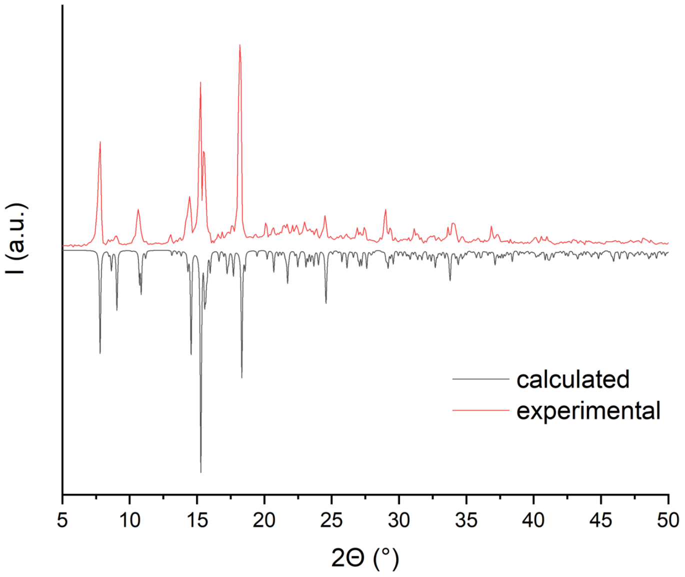

Figure S10. Powder X-ray diffraction plots for the coordination polymer **2**.

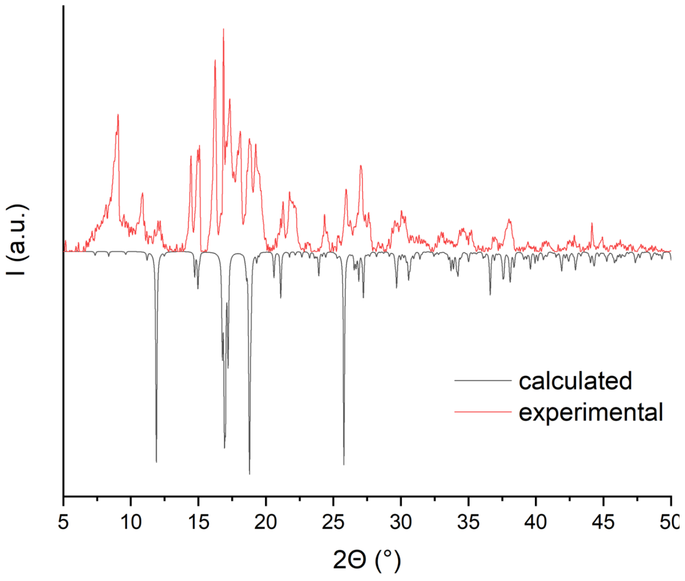

Figure S11. Powder X-ray diffraction plots for the coordination polymer **3**.

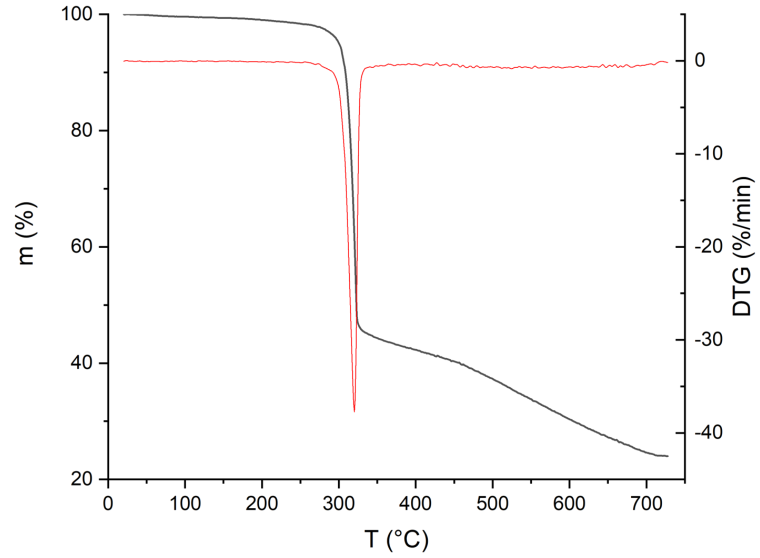

Figure S12. TG and DTA curves for the coordination polymer **1**.

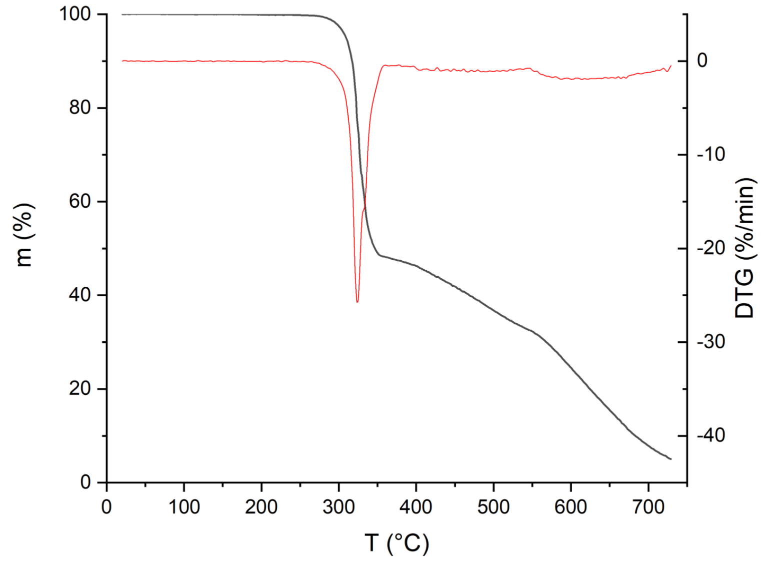

Figure S13. TG and DTA curves for the coordination polymer **2**.

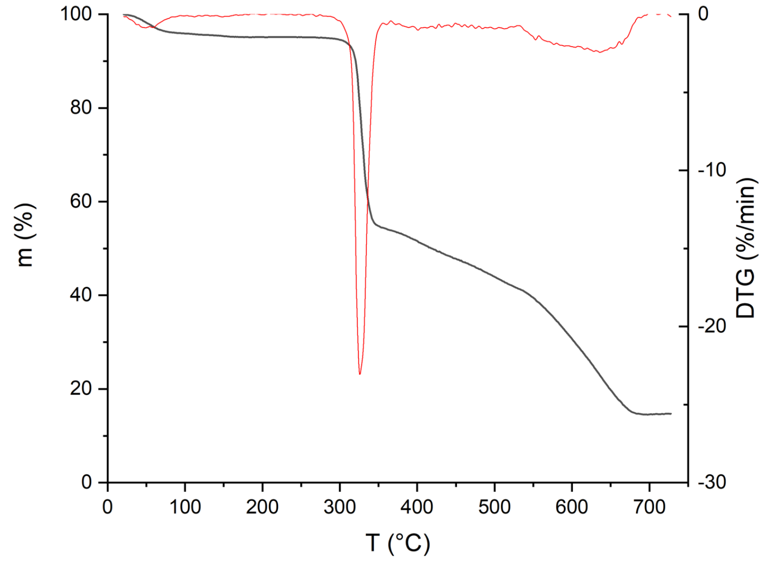

Figure S14. TG and DTA curves for the coordination polymer **3**.

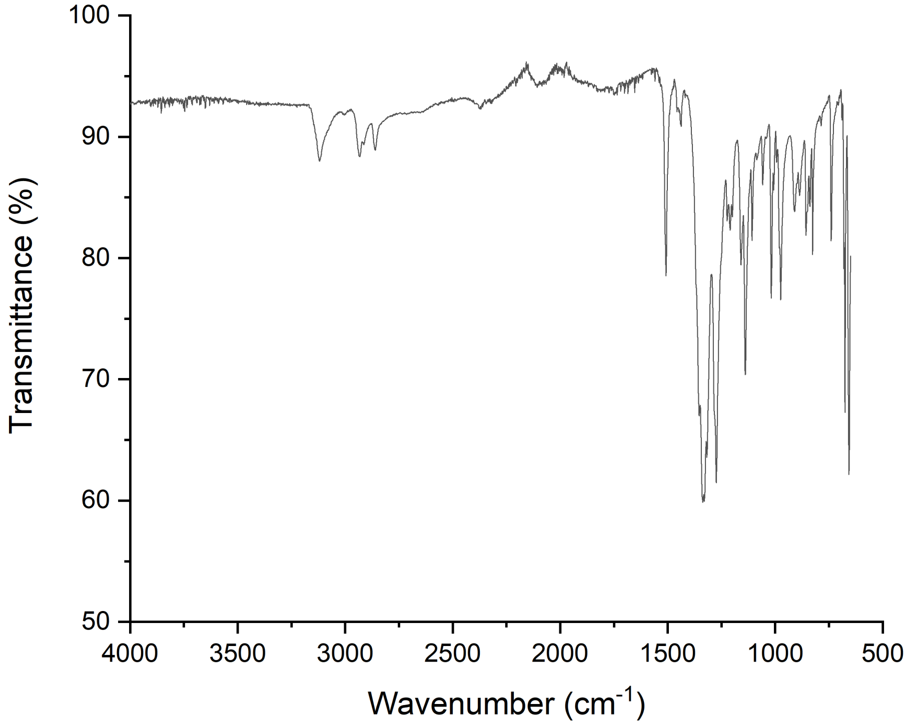

Figure S15. FT-IR spectrum of the coordination polymer **1**.

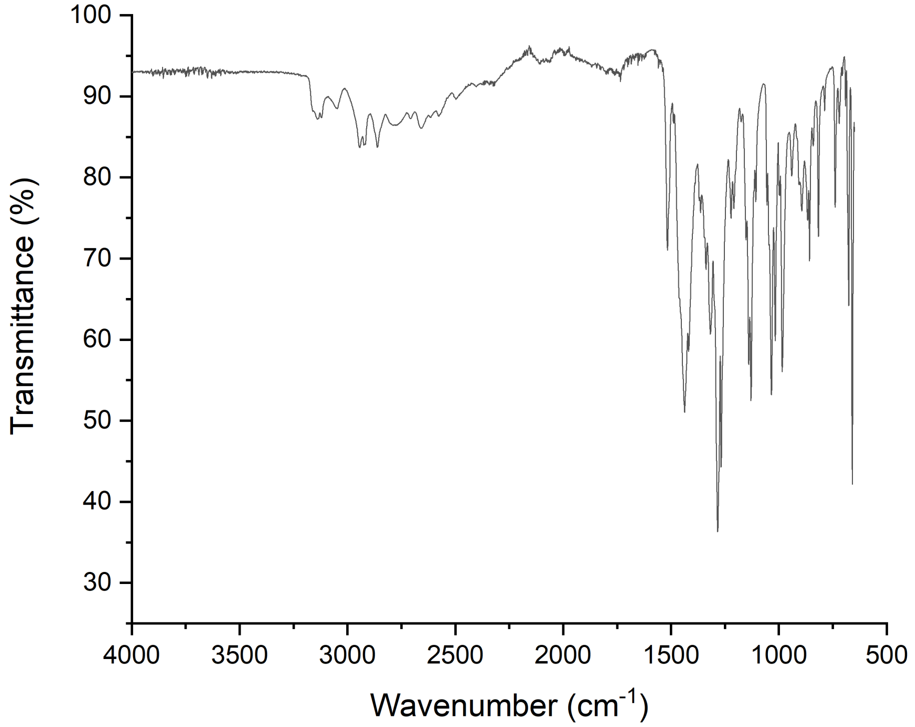

Figure S16. FT-IR spectrum of the coordination polymer **2**.

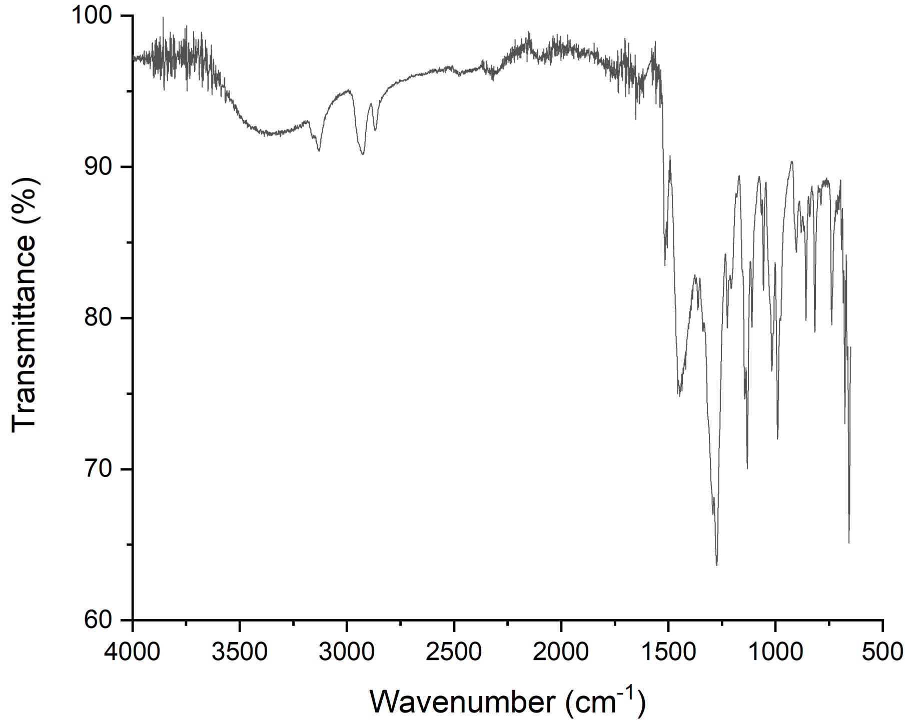

Figure S17. FT-IR spectrum of the coordination polymer **3**.

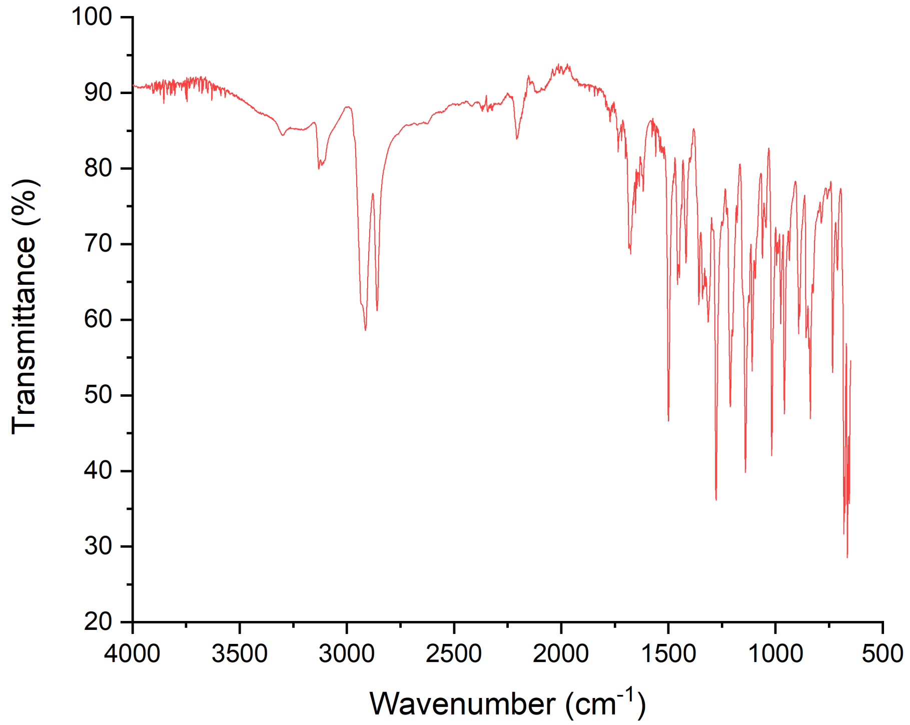

Figure S18. FT-IR spectrum of the ligand **L**.

Table S1. Interatomic distances, angles and geometrical criteria for nitrate coordination in compounds **1**-**3**.

Compound **1**				
d_1 (Ag1–O14), Å	2.640	d_2-d_1, Å	1.204 > 0.6	unidentate
d_2 (Ag1–O15), Å	3.844	A_1-A_2, °	61.46 > 28	
d_3 (Ag1–N18), Å	3.508	d_3-d_2, Å	-0.336 < 0.1	
A_1 (Ag1-O14-N18),°	126.56	A_3, °	123.92 < 162	
A_2 (Ag1-O15-N18),°	65.10			
A_3 (Ag1-N18-O16),°	123.92			
d_1 (Ag1–O11), Å	2.852	d_2-d_1, Å	1.105 > 0.6	unidentate
d_2 (Ag1–O13), Å	3.957	A_1-A_2, °	59.85 > 28	
d_3 (Ag1–N17), Å	3.827	d_3-d_2, Å	-0.13 < 0.1	
A_1 (Ag1-O11-N17),°	134.77	A_3, °	155.06 < 162	
A_2 (Ag1-O13-N17),°	74.92			
A_3 (Ag1-N17-O12),°	155.06			
Compound **2**				
d_1 (Cd1–O12), Å	2.524	d_2-d_1, Å	0.103 < 0.3	bidentate
d_2 (Cd1–O11), Å	2.627	A_1-A_2, °	4.87 < 14	
d_3 (Cd1–N3), Å	2.967	d_3-d_2, Å	0.34 > 0.2	
A_1 (Cd1-O12-N3),°	97.89	A_3, °	171.84 > 168	
A_2 (Cd1-O11-N3),°	93.02			
A_3 (Cd1-N3-O13),°	171.84			
d_1 (Cd1–O21), Å	2.388	d_2-d_1, Å	0.860 > 0.6	unidentate
d_2 (Cd1–O22), Å	3.248	A_1-A_2, °	41.97 > 28	
d_3 (Cd1–N4), Å	3.220	d_3-d_2, Å	-0.028 < 0.1	
A_1 (Cd1-O21-N4),°	119.67	A_3, °	156.73 < 162	
A_2 (Cd1-O22-N4),°	77.70			
A_3 (Cd1-N4-O23),°	156.73			
Compound **3**				
d_1 (Cd1–O11), Å	2.385	d_2-d_1, Å	0.217 < 0.3	bidentate
d_2 (Cd1–O12), Å	2.602	A_1-A_2, °	11.50 < 14	
d_3 (Cd1–N51), Å	2.917	d_3-d_2, Å	0.315 > 0.2	
A_1 (Cd1-O11-N51),°	103.03	A_3, °	175.74 > 168	
A_2 (Cd1-O12-N51),°	91.53			
A_3 (Cd1-N51-O13),°	175.74			
d_1 (Cd1–O15), Å	2.448	d_2-d_1, Å	0.052 < 0.3	bidentate
d_2 (Cd1–O14), Å	2.500	A_1-A_2, °	-2 < 14	
d_3 (Cd1–N52), Å	2.861	d_3-d_2, Å	0.361 > 0.2	
A_1 (Cd1-O14-N52),°	93.97	A_3, °	176.72 > 168	
A_2 (Cd1-O15-N52),°	95.97			
A_3 (Cd1-N52-O16),°	176.72			
d_1 (Cd2–O22), Å	2.330	d_2-d_1, Å	0.178 < 0.3	bidentate
d_2 (Cd2–O21), Å	2.508	A_1-A_2, °	7.99 < 14	
d_3 (Cd2–N72), Å	2.828	d_3-d_2, Å	0.320 > 0.2	
A_1 (Cd2-O22-N72),°	100.23	A_3, °	172.38 > 168	
A_2 (Cd2-O21-N72),°	92.24			
A_3 (Cd2-N72-O23),°	172.38			
d_1 (Cd2–O25), Å	2.401	d_2-d_1, Å	0.035 < 0.3	bidentate
d_2 (Cd2–O24), Å	2.436	A_1-A_2, °	0.95 < 14	
d_3 (Cd2–N71), Å	2.823	d_3-d_2, Å	0.387 > 0.2	
A_1 (Cd2-O25-N71),°	96.00	A_3, °	173.48 > 168	
A_2 (Cd2-O24-N71),°	95.05			
A_3 (Cd2-N71-O26),°	173.48			

Table S2. Continuous shape measures criteria for seven-coordinated cadmium centers in compounds **2** and **3**.

Compound	S(PBPY)	S(COC)	S(CTPR)
Cd1 in **2**	1.279	7.553	5.924
Cd1 in **3**	3.927	7.726	6.245
Cd2 in **3**	2.570	8.910	7.211
