# Supplementary material for: Synthesis, Crystal Structure, and Luminescence of Cadmium(II) and Silver(I) Coordination Polymers Based on 1,3-Bis(1,2,4-triazol-1-yl)adamantane
Source: Molecules. 2021 Sep 5;26(17):5400. doi: 10.3390/molecules26175400 (PMC8434004; doi:10.3390/molecules26175400)

checkCIF/PLATON report

Structure factors have been supplied for datablock(s) pot_ag011_1

THIS REPORT IS FOR GUIDANCE ONLY. IF USED AS PART OF A REVIEW PROCEDURE FOR PUBLICATION, IT SHOULD NOT REPLACE THE EXPERTISE OF AN EXPERIENCED CRYSTALLOGRAPHIC REFEREE.

No syntax errors found. CIF dictionary Interpreting this report

Datablock: pot_ag011_1

```
Bond precision:  C-C = 0.0093 A         Wavelength=0.71073

Cell:           a=13.3900(4)    b=9.7545(3)        c=13.0518(4)
                alpha=90        beta=110.436(1)    gamma=90
Temperature:    298 K

                Calculated                Reported
Volume          1597.44(9)                1597.44(8)
Space group     P 21/c                    P 1 21/c 1
Hall group      -P 2ybc                   -P 2ybc
Moiety formula  C14 H18 Ag N7 O3          C14 H18 Ag N7 O3
Sum formula     C14 H18 Ag N7 O3          C14 H18 Ag N7 O3
Mr              440.22                    440.22
Dx,g cm-3       1.831                     1.830
Z               4                         4
Mu (mm-1)       1.294                     1.294
F000            888.0                     888.0
F000'           884.70
h,k,lmax        15,11,15                  15,11,15
Nref            2630                      2630
Tmin,Tmax       0.883,0.937               0.688,0.745
Tmin'           0.845

Correction method= # Reported T Limits: Tmin=0.688 Tmax=0.745
AbsCorr = MULTI-SCAN

Data completeness= 1.000          Theta(max)= 24.405

R(reflections)= 0.0549( 1961)     wR2(reflections)= 0.1478( 2630)

S = 1.047                  Npar= 263
```

The following ALERTS were generated. Each ALERT has the format
test-name_ALERT_alert-type_alert-level.
Click on the hyperlinks for more details of the test.

Alert level B

```
PLAT232_ALERT_2_B Hirshfeld Test Diff (M-X)  Ag1      --N15_b    .       12.7 s.u.
```

Author Response: There is no doubt that assignment of Ag and N atoms is correct, because the reagents containing these elements were used for the synthesis. Uncorrelated anisotropic parameters of Ag1 and N15 can be due to low rigidity of the corresponding bond or a slight disorder of the Ag atom.

Alert level C

```
THETM01_ALERT_3_C  The value of sine(theta_max)/wavelength is less than 0.590
            Calculated sin(theta_max)/wavelength =    0.5814
PLAT094_ALERT_2_C Ratio of Maximum / Minimum Residual Density ....       2.47 Report
PLAT234_ALERT_4_C Large Hirshfeld Difference Ag1      --O14      .       0.17 Ang.
PLAT241_ALERT_2_C High   'MainMol' Ueq as Compared to Neighbors of        N12 Check
PLAT242_ALERT_2_C Low    'MainMol' Ueq as Compared to Neighbors of        Ag1 Check
PLAT342_ALERT_3_C Low Bond Precision on  C-C Bonds ...............    0.00933 Ang.
PLAT906_ALERT_3_C Large K Value in the Analysis of Variance ......      2.404 Check
PLAT971_ALERT_2_C Check Calcd Resid. Dens.  0.85A   From Ag1             2.19 eA-3
```

Alert level G

```
PLAT002_ALERT_2_G Number of Distance or Angle Restraints on AtSite          8 Note
PLAT003_ALERT_2_G Number of Uiso or Uij Restrained non-H Atoms ...          8 Report
PLAT004_ALERT_5_G Polymeric Structure Found with Maximum Dimension          3 Info
PLAT083_ALERT_2_G SHELXL Second Parameter in WGHT  Unusually Large       6.71 Why ?
PLAT172_ALERT_4_G The CIF-Embedded .res File Contains DFIX Records          2 Report
PLAT173_ALERT_4_G The CIF-Embedded .res File Contains DANG Records          6 Report
PLAT174_ALERT_4_G The CIF-Embedded .res File Contains FLAT Records          2 Report
PLAT187_ALERT_4_G The CIF-Embedded .res File Contains RIGU Records          2 Report
PLAT301_ALERT_3_G Main Residue  Disorder ..............(Resd  1  )        16% Note
PLAT860_ALERT_3_G Number of Least-Squares Restraints .............         50 Note
PLAT909_ALERT_3_G Percentage of I>2sig(I) Data at Theta(Max) Still        35% Note
PLAT978_ALERT_2_G Number C-C Bonds with Positive Residual Density.          1 Info
```

---

0 **ALERT level A** = Most likely a serious problem - resolve or explain
1 **ALERT level B** = A potentially serious problem, consider carefully
8 **ALERT level C** = Check. Ensure it is not caused by an omission or oversight
12 **ALERT level G** = General information/check it is not something unexpected

0 ALERT type 1 CIF construction/syntax error, inconsistent or missing data
9 ALERT type 2 Indicator that the structure model may be wrong or deficient
6 ALERT type 3 Indicator that the structure quality may be low
5 ALERT type 4 Improvement, methodology, query or suggestion
1 ALERT type 5 Informative message, check

It is advisable to attempt to resolve as many as possible of the alerts in all categories. Often the minor alerts point to easily fixed oversights, errors and omissions in your CIF or refinement strategy, so attention to these fine details can be worthwhile. In order to resolve some of the more serious problems it may be necessary to carry out additional measurements or structure refinements. However, the purpose of your study may justify the reported deviations and the more serious of these should normally be commented upon in the discussion or experimental section of a paper or in the "special_details" fields of the CIF. checkCIF was carefully designed to identify outliers and unusual parameters, but every test has its limitations and alerts that are not important in a particular case may appear. Conversely, the absence of alerts does not guarantee there are no aspects of the results needing attention. It is up to the individual to critically assess their own results and, if necessary, seek expert advice.

Publication of your CIF in IUCr journals

A basic structural check has been run on your CIF. These basic checks will be run on all CIFs submitted for publication in IUCr journals (*Acta Crystallographica*, *Journal of Applied Crystallography*, *Journal of Synchrotron Radiation*); however, if you intend to submit to *Acta Crystallographica Section C* or *E* or *IUCrData*, you should make sure that full publication checks are run on the final version of your CIF prior to submission.

Publication of your CIF in other journals

Please refer to the *Notes for Authors* of the relevant journal for any special instructions relating to CIF submission.

PLATON version of 22/03/2021; check.def file version of 19/03/2021

Datablock pot_ag011_1 - ellipsoid plot

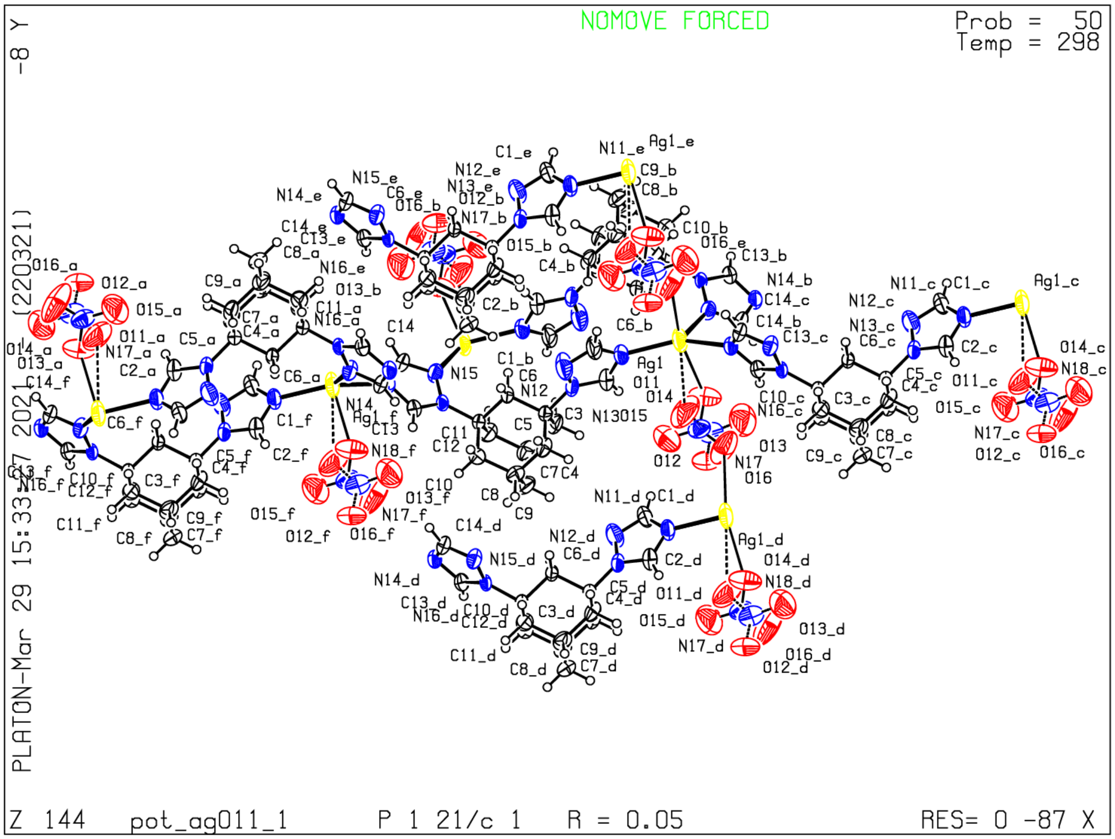

Supplement: Supplementary file 1 [file Compound1_CheckCIF.pdf › Compound1_CheckCIF.pdf]
