# Supplementary material for: Synthesis, Crystal Structure, and Luminescence of Cadmium(II) and Silver(I) Coordination Polymers Based on 1,3-Bis(1,2,4-triazol-1-yl)adamantane
Source: Molecules. 2021 Sep 5;26(17):5400. doi: 10.3390/molecules26175400 (PMC8434004; doi:10.3390/molecules26175400)

checkCIF/PLATON report

Structure factors have been supplied for datablock(s) pot_cd520

THIS REPORT IS FOR GUIDANCE ONLY. IF USED AS PART OF A REVIEW PROCEDURE FOR PUBLICATION, IT SHOULD NOT REPLACE THE EXPERTISE OF AN EXPERIENCED CRYSTALLOGRAPHIC REFEREE.

No syntax errors found. CIF dictionary Interpreting this report

Datablock: pot_cd520

```
Bond precision:  C-C = 0.0036 A          Wavelength=0.71073

Cell:            a=11.3904(13)     b=13.4621(12)      c=20.904(2)
                 alpha=90          beta=93.070(4)     gamma=90
Temperature:     150 K

                Calculated                  Reported
Volume          3200.8(6)                   3200.7(6)
Space group     P 21/n                      P 1 21/n 1
Hall group      -P 2yn                      -P 2yn
Moiety formula  C28 H36 Cd N14 O6           C28 H36 Cd N14 O6
Sum formula     C28 H36 Cd N14 O6           C28 H36 Cd N14 O6
Mr              777.12                      777.11
Dx,g cm-3       1.613                       1.613
Z               4                           4
Mu (mm-1)       0.749                       0.749
F000            1592.0                      1592.0
F000’           1589.33
h,k,lmax        13,16,25                    13,16,25
Nref            6097                        6089
Tmin,Tmax       0.931,0.963                 0.630,0.745
Tmin’           0.741

Correction method= # Reported T Limits: Tmin=0.630 Tmax=0.745
AbsCorr = MULTI-SCAN

Data completeness= 0.999          Theta(max)= 25.695

R(reflections)= 0.0315( 4920)     wR2(reflections)= 0.0720( 6089)

S = 1.007                   Npar= 442
```

```
The following ALERTS were generated. Each ALERT has the format
       test-name_ALERT_alert-type_alert-level.
Click on the hyperlinks for more details of the test.
```

Alert level B

```
PLAT232_ALERT_2_B Hirshfeld Test Diff (M-X)  Cd1      --O12      .       11.6 s.u.
```

Author Response: There is no doubt that assignment of Cd and O atoms is correct, because the reagents containing these elements were used for the synthesis. Uncorrelated anisotropic parameters of Cd1 and O12 can be due to low rigidity of the corresponding bond which has mailny ionic character.

Alert level C

```
PLAT213_ALERT_2_C Atom O13             has ADP max/min Ratio .....        3.4 prolat
PLAT220_ALERT_2_C NonSolvent   Resd 1  O   Ueq(max)/Ueq(min) Range        3.1 Ratio
PLAT230_ALERT_2_C Hirshfeld Test Diff for    O23      --N4       .        5.5 s.u.
PLAT241_ALERT_2_C High   'MainMol' Ueq as Compared to Neighbors of        O11 Check
PLAT242_ALERT_2_C Low    'MainMol' Ueq as Compared to Neighbors of         N3 Check
PLAT911_ALERT_3_C Missing FCF Refl Between Thmin & STh/L=    0.600          5 Report
PLAT976_ALERT_2_C Check Calcd Resid. Dens.  1.03A   From O13            -0.41 eA-3
```

Alert level G

```
PLAT004_ALERT_5_G Polymeric Structure Found with Maximum Dimension          1 Info
PLAT232_ALERT_2_G Hirshfeld Test Diff (M-X)  Cd1      --O11      .       18.1 s.u.
```

Author Response: There is no doubt that assignment of Cd and O atoms is correct, because the reagents containing these elements were used for the synthesis. Uncorrelated anisotropic parameters of Cd1 and O12 can be due to low rigidity of the corresponding bond which has mailny ionic character.

```
PLAT794_ALERT_5_G Tentative Bond Valency for Cd1       (II)      .       2.03 Info
PLAT804_ALERT_5_G Number of ARU-Code Packing Problem(s) in PLATON           1 Info
PLAT912_ALERT_4_G Missing # of FCF Reflections Above STh/L=  0.600          3 Note
PLAT941_ALERT_3_G Average HKL Measurement Multiplicity ..........         3.1 Low
PLAT978_ALERT_2_G Number C-C Bonds with Positive Residual Density.          7 Info
```

```
   0 ALERT level A = Most likely a serious problem - resolve or explain
   1 ALERT level B = A potentially serious problem, consider carefully
   7 ALERT level C = Check. Ensure it is not caused by an omission or oversight
   7 ALERT level G = General information/check it is not something unexpected

   0 ALERT type 1 CIF construction/syntax error, inconsistent or missing data
   9 ALERT type 2 Indicator that the structure model may be wrong or deficient
   2 ALERT type 3 Indicator that the structure quality may be low
   1 ALERT type 4 Improvement, methodology, query or suggestion
   3 ALERT type 5 Informative message, check
```

It is advisable to attempt to resolve as many as possible of the alerts in all categories. Often the minor alerts point to easily fixed oversights, errors and omissions in your CIF or refinement strategy, so attention to these fine details can be worthwhile. In order to resolve some of the more serious problems it may be necessary to carry out additional measurements or structure refinements. However, the purpose of your study may justify the reported deviations and the more serious of these should normally be commented upon in the discussion or experimental section of a paper or in the "special_details" fields of the CIF. checkCIF was carefully designed to identify outliers and unusual parameters, but every test has its limitations and alerts that are not important in a particular case may appear. Conversely, the absence of alerts does not guarantee there are no aspects of the results needing attention. It is up to the individual to critically assess their own results and, if necessary, seek expert advice.

Publication of your CIF in IUCr journals

A basic structural check has been run on your CIF. These basic checks will be run on all CIFs submitted for publication in IUCr journals (*Acta Crystallographica*, *Journal of Applied Crystallography*, *Journal of Synchrotron Radiation*); however, if you intend to submit to *Acta Crystallographica Section C* or *E* or *IUCrData*, you should make sure that full publication checks are run on the final version of your CIF prior to submission.

Publication of your CIF in other journals

Please refer to the *Notes for Authors* of the relevant journal for any special instructions relating to CIF submission.

PLATON version of 22/03/2021; check.def file version of 19/03/2021

Datablock pot_cd520 - ellipsoid plot

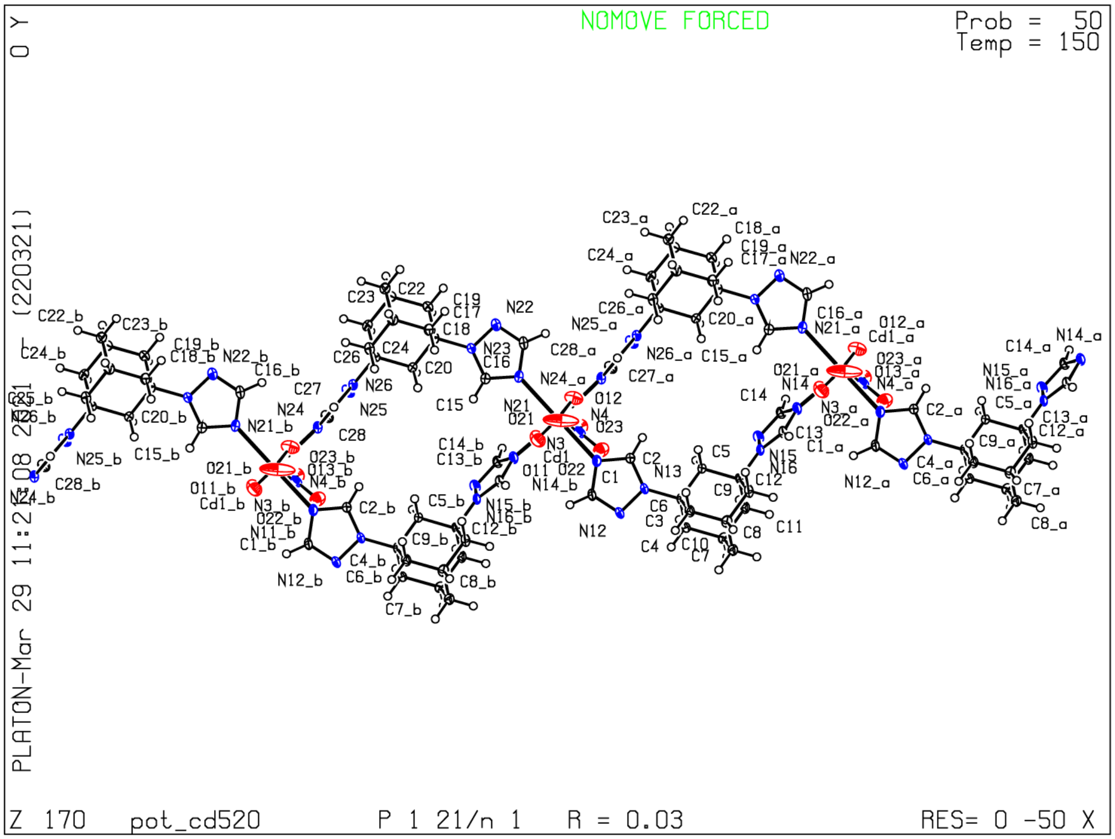

Supplement: Supplementary file 1 [file Compound2_CheckCIF.pdf › Compound2_CheckCIF.pdf]
